# Prevalence of demoralization and depressive symptoms in a sample of patients with supraventricular tachyarrhythmias: preliminary results

**DOI:** 10.3389/fpsyt.2024.1355031

**Published:** 2024-07-25

**Authors:** Tommaso Accinni, Annalisa Maraone, Alessio Bonucci, Andrea D’Amato, Carlo Lavalle, Francesco Saverio Bersani, Paolo Severino, Massimo Pasquini

**Affiliations:** ^1^ Department of Human Neurosciences, Faculty of Medicine and Dentistry, Sapienza University of Rome, Rome, Italy; ^2^ Department of Clinical, Internal, Anesthesiology and Cardiovascular Sciences, Sapienza University of Rome, Rome, Italy

**Keywords:** demoralization, depression, depressive symptoms, supraventricular tachyarrhythmias, atrial fibrillation, atrial flutter, paroxysmal supraventricular tachycardia, mental health

## Abstract

**Introduction:**

Supraventricular tachyarrhythmias (ST) are the most common cardiac arrhythmias. Little is known about the potential impact of demoralization, which is considered as partially distinct from depression, on the course of ST. A correct assessment of both depressive symptoms and demoralization appears relevant for the treatment of these cardiac diseases, potentially influencing their course.

**Methods:**

The sample consisted of 110 subjects affected by different ST, such as atrial fibrillation (AF), atrial flutter (AFL) and paroxysmal supraventricular tachycardia (PSVT). They all underwent a psychiatric evaluation; the Italian version of 9-item Patient Health Questionnaire (PHQ-9) and the Italian version of Demoralization Scale (DS) were administered. Descriptive statistics, pairwise comparisons, and correlational analysis have been implemented.

**Results:**

26 individuals (23.6%) presented high levels of demoralization. Of these, 20 (76.9%) had a diagnosis of AF and six patients (23.1%) received a diagnosis of other ST. No differences in demoralization levels resulted in regard of sex, cardiac diagnoses and anticoagulant therapies. Amongst people with high levels of demoralization, 13 (50%) received no formal psychiatric diagnosis, and 12 (46.2%) showed moderate/severe depressive symptoms. Demoralization levels and PHQ-9 scores showed a significant positive correlation in the whole sample (r=0.550, p<0.001).

**Discussion:**

The present study found that in a sample of patients suffering from ST, high levels of demoralization were more frequent than clinically relevant depressive symptoms. We propose that demoralization and depression show partially distinguished psychopathological features, potentially associated with different therapeutic trajectories.

## 1 Introduction

Supraventricular tachyarrhythmias (ST), such as atrial fibrillation (AF), atrial flutter (AFL) and paroxysmal supraventricular tachycardia (PSVT) are a widespread group of diseases, representing the most common type of cardiac arrhythmias (1), with a clinical relevance in Italy as well (1), associated with a significantly increased risk of thromboembolic events, hospitalization and mortality worldwide (2). Recent research has investigated the potential impact of mental states, particularly of depression, on the short and long-term course of cardiac diseases. Anxiety and depressive symptoms seem to be bidirectionally associated with arrhythmias (3). On one hand, the burden of cardiorespiratory symptoms (chest pain, palpitations shortness of breath, dizziness, general weakness) can lead to sustained psychological stress. On the other hand, sustained psychological stress can worsen the clinical course of tachyarrhythmias, influencing compliance to treatment and clinical outcomes by increasing the possibility of developing severe cardiovascular consequences such as ischemic stroke and intracranial bleeding (4–7).

Despite its clinical relevance in consultation-liaison psychiatry, the construct of demoralization has been poorly investigated in chronic heart diseases. Originally defined by Frank (8) as a stressful state of existential suffering denoting a persistent failure of coping strategies related to medical settings, demoralization has recently been considered as a psychopathological dimension partially distinct from depression, possibly leading to a specific syndrome (often defined as “Demoralization Syndrome”, DS) (9). Demoralization seems to be characterized by specific psychopathological features such as helplessness, hopelessness, sense of failure and a self-perceived incapacity to react appropriately in stressful situations. DS differs from major depression especially for the low impact of anhedonia on the clinical condition (10). However, since demoralization and major depression often co-occur, a risk is that the first one may be partially unrecognized or mistaken for the latter (11). Importantly, as these disorders have different core symptoms, they could require different interventions (10, 12). Previous studies in patients with oncological diseases or with other chronic illnesses have shown that depressive symptoms and clinically-relevant demoralization were related to a poorer prognosis (13–16) and that demoralization, compared to depression, is associated with a higher suicide risk (17). Furthermore, adverse health outcomes, re-hospitalization and lower compliance to medication have been reported in myocardial infarction survivors and heart transplant patients with DS (18, 19).

Overall, a correct assessment of both depression and demoralization seems to be relevant for the treatment and long-term management of chronic diseases. With the present pilot study, we aimed at preliminarily describing the degree of demoralization in a sample of outpatients and hospitalized subjects affected by ST.

## 2 Materials and methods

### 2.1 Sample

This preliminary work emerges in the context of a broader study approved by the Ethical Committee of Policlinico Umberto I Hospital (protocol 0950/2022) which investigates psychopathology in chronic heart diseases. A total of 110 participants with ST were included in the current study, recruited from inpatient and outpatient Cardiovascular Disease Unit of Policlinico Umberto I Hospital of Rome. All subjects gave informed consent. Inclusion criteria for the present study were: 1) diagnosis of ST (AF, AFL or PSVT), according to European Society of Cardiology guidelines (20, 21); 2) age ≥ 18 years. Exclusion criteria were: 1) previous diagnosis of schizophrenia spectrum disorders (due to potential impairments in cognition, insight and affective regulation, which may interfere with the study objectives), 2) history of substance or alcohol related disorders; 3) current major neurocognitive disorders or intellectual disability; 4) cancer comorbidity; 5) impossibility to give a written informed consent. Trained staff collected sociodemographic data and clinical variables during patients’ routine cardiovascular assessments.

### 2.2 Psychiatric assessments

All patients underwent psychiatric evaluation involving a clinical interview based on the criteria of the Diagnostic and Statistical Manual of Mental Disorders 5^th^ Edition (DSM5) (22) performed by a psychiatrist. Validated self-report questionnaires have been administered to assess depressive and demoralization symptoms, respectively the Italian version of 9-item Patient Health Questionnaire (PHQ-9) and the Italian version of Demoralization Scale (DS) (22–24).

The PHQ-9 consists of 9 items investigating the depressive symptoms as described in the DSM-5, each of them rated on a four-point Likert scale (ranging from 0 to 3), covering a time frame of the past two weeks. A score ≥10 has been used for the screening of depressive symptoms (absent or mild depressive symptoms vs moderate or severe depressive symptoms) (22).

The DS consists of 24 items covering 5 dimensions (thus having 5 subscales): *Loss of meaning and purpose, Dysphoria, Disheartenment, Helplessness, Sense of failure* (9). Every item is rated on a five-point Likert scale (ranging from 0 to 4). The original version of the scale entailed a total global score obtained by adding the rating of each singular item. In the original validation study by Kissane et al. (23), a global average score of 30.82 ± 17.73 was observed in the recruited cancer sample, and a total global score >30 was used to define high levels of demoralization (this cutoff was also used in a study on the Italian validation of DS (24). In the present work we maintained the above-mentioned score as cut-off in relation to our research purposes.

### 2.3 Statistical analyses

As this is a preliminary report aimed at introducing the study of demoralization in the context of patients with ST, we have mainly focused on descriptive statistics. We have also implemented preliminary inferential analyses: we tested pairwise comparisons (Student *t-test* or ANalysis Of VAriance – ANOVA) to investigate between-group differences in demoralization levels, and correlational analyses (Pearson Correlation) to test the association between demoralization and PHQ-9, after controlling for normal distribution of data (values of skewness and kurtosis between -2 and +2 (25) and after controlling for homogeneity of variances by means of Levene’s Test for equality of variances both for Student’s t-test and ANOVA; Chi-square test was used to compare categorical variables. Statistical significance was set at *p* < 0.05 for all analyses. We used the SPSS 27.0 version (Statistical Package for the Social Sciences, IBM Co., Armonk, New York, 2017) for all statistical analyses.

## 3 Results

The demographic and clinical characteristics of the sample are presented in **Table 1**.

**Table 1:** Demographical and clinical variables of the recruited sample.

Mean age ± SD	76.05 ± 10.69				
Sex: N = female (%)	40 (36.4%)				
Marital status	75 (68.2%) married	6 (5.5%) divorced	24 (21.8%) widowers	5 (4.5%) unmarried	
ST diagnosis and characterization	89 (80.9%) with AF			21 (19.1%) with other ST (AFL and PSVT)	
	52 (47.3%) with paroxysmal AF	8 (7.3%) with persistent AF	29 (26.4%) with permanent AF		
Cardiological characteristics	32 (29.1%) CCS	58 (52.7%) DYS	27 (24.5%) T2D	71 (64.5%) CHF	88 (80%) HYP
ACs therapy	78 (70.9%) on DOACs	24 (21.8%) on VKA	8 (7.3%) with no ACs		

SD, standard deviation; ST, supraventricular tachyarrhythmias; AF, atrial fibrillation; AFL, atrial flutter; PSVT, paroxysmal supraventricular tachycardia; ACs, anticoagulants; DOACs, direct-acting oral anticoagulants; VKA, vitamin K antagonist; CCS, chronic coronary syndrome; DYS, dyslipidemia; T2D, type II diabetes mellitus; CHF, chronic heart failure; HYP, hypertension.

Looking at the DS, the mean score for the recruited group was 22.65 ± 11.94; considering the DS subscales, the mean score for *Loss of meaning and purpose* was 2.17 ± 3.03, for *Dysphoria* was 4.45 ± 3.44, for *Disheartenment* was 6.05 ± 4.08, for *Helplessness* was 2.71 ± 2.60, and for *Sense of failure* was 7.28 ± 4.90.

Concerning the demoralization severity, as already discussed we considered a score >30 to define the presence of high levels of demoralization (20): thereby, in the whole sample 26 individuals (23.6%) presented high levels of demoralization. Among the 26 individuals with high levels of demoralization, nine (34.6%) were females (while 17 were males); 22 (84.6%) were hospitalized (while 4 were outpatients); 20 (76.9%) had a diagnosis of AF (of which 13 paroxysmal AF, one patient persistent AF, six patients permanent AF) while six patients received a diagnosis of other ST).

Across all recruited subjects, no significant differences in demoralization levels were found between hospitalized people and outpatients (t=-1.491; p=0.139), and between females and males (t=-0.619; p=0.537); no differences in demoralization levels were observed in regard of different cardiovascular diagnoses and anticoagulant therapy (F=1.019; p=0.388 and F=0.320 p=0.727 respectively). Similarly, using categorical variables we did not find significantly different prevalence of individuals with high DS between females and males (χ_2_ = 0.045; p=0.832), between people with different cardiovascular diagnosis (χ_2_ = 1.026; p=0.795), and between people using different anticoagulant therapies (χ_2_ = 3.403; p=0.182).

About psychiatric comorbidity in the whole sample, 27 (24.5%) patients received a formal psychiatric diagnosis: in particular, one (0.9%) had a major depressive disorder (MDD), one (0.9%) had a bipolar disorder (BD), one (0.9%) had a post-traumatic stress disorder (PTSD), eight (7.3%) had an anxiety disorder (AD), and 16 (14.5%) had an adjustment disorder (ADD). We found significant differences in demoralization symptoms between patients who received a formal psychiatric diagnosis and patients without a psychiatric diagnosis (t=-3.518; p<0.001), with higher scores in the former group (29.3 ± 13.4) compared to the second (20.5 ± 10.6).

Regarding the 26 individuals with high levels of demoralization, 13 (50%) received no formal psychiatric diagnosis, three (11.5%) had a diagnosis of AD, eight (30.8%) had a diagnosis of ADD, while the remaining subjects received other psychiatric diagnoses (one had a BD and one had a PTSD, counting for 3.8% each).

Looking at the PHQ-9 scale for the entire sample, 14 individuals (12.7%) showed a moderate/severe degree of depressive symptoms, while 96 individuals showed none/mild depressive symptoms.

Examining the overlap between depressive and demoralization symptoms, we found that 12 patients with high levels of demoralization (i.e., 46.2% of patients with high DS) showed moderate/severe depressive symptoms, while 14 patients with high levels of demoralization (i.e. 53.8% of patients with high DS) showed none/mild depressive symptoms.

Demoralization levels and PHQ-9 scores showed a significant positive correlation in the whole sample (r=0.550, p<0.001).

## 4 Discussion

To the best of our knowledge, this is the first report exploring demoralization in a sample of patients with ST. The results of the present preliminary study are consistent with previous evidence which observed depression among patients with ST (26, 27), and contribute to extend the knowledge on the filed by focusing on the construct of demoralization.

In our sample, 23.6% of patients showed high levels of demoralization. This finding is consistent with a recent systematic review by Gan et al. (2022) on oncological patients (24). Notably, in our sample 53.8% of participants with high levels of demoralization showed none/mild depressive symptoms, likely representing a group of clinical subjects who did not meet formal categorical criteria for a psychiatric diagnosis. A high prevalence of Adjustment Disorder (28.6%) was observed amongst patients with high levels of demoralization, potentially reflecting a common inadequacy to cope with illness related stressors (28).

Depressive and demoralization symptoms might be associated with high arrhythmia variability regarding their nature, frequency, timing of onset and duration. For example, the fear of new episodes in individuals affected by paroxysmal AF may induce an anxious state which may become more limiting than the cardiac condition itself (29). Similarly, unpredictable disease manifestations and existential distress could explain the high rate of demoralized patients with paroxysmal AF in our study. The number of hospitalizations and treatments are well-established factors associated with higher levels of demoralization (29). This observation was partially confirmed by our data indicating that among the 26 individuals with high levels of demoralization 84.6% (n=22) were hospitalized while 15,4% (n=4) were outpatients, although our results did not underline significant differences in the levels of demoralization between inpatients and outpatients (this possibly reflecting the connection between DS and the chronicity of dysrhythmia rather than the acute cardiac event).

Starting from our observations suggesting a larger amount of patients with high levels of demoralization in a sample of patients with ST, compared to a smaller amount of patients with moderate/severe depressive symptoms, we postulate that a differential diagnosis between demoralization and depression can be relevant, especially because demoralization appears poorly responsive to standard antidepressant therapies while it appears to be more sensitive to cognitive or existential psychotherapy (30, 31). Moreover, certain antidepressants can be associated with cardiovascular side effects such as bleeding, hyponatremia, pharmacokinetic interactions and increased proarrhythmic risk due to QT prolongation (32), and therefore it is important to limit their use in patients with cardiac arrhythmias to those cases in which it is strictly necessary. Overall, it is possible that the existence of distinguished psychopathological features between demoralization and depressive symptoms can potentially be associated with differences in treatment trajectories.

Certain limitations should be taken into consideration in the interpretation of study results. Firstly, our work is a pilot study with the aim to describe demoralization in ST patients. The statistical analyses are limited to preliminary data available at the moment, while other variables important to understand and quantify the severity of dysrhythmias/cardiological dysfunctions are currently missing (blood tests, electrocardiogram, echocardiogram, re-hospitalizations, acute events) and need to be taken into consideration in future studies on the topic. Secondly, the study is cross-sectional: longitudinal studies would be needed to explore the potential impact of demoralization on the short and long-term course of tachyarrhythmias. Thirdly, our work did not include a control group that could have provided additional information and comparisons of demoralization levels amongst different clinical samples.

In conclusion, the present preliminary study contributes to shed some light on the complex relationships between demoralization, depressive symptoms and certain arrhythmias, with further studies needed to corroborate the findings and to fully investigate the potential impact of demoralization on longitudinal course of tachyarrhythmias.

## Data availability statement

The raw data supporting the conclusions of this article will be made available by the authors, without undue reservation.

## Ethics statement

The studies involving humans were approved by Ethical Committee of Policlinico Umberto I Hospital (protocol 0950/2022). The studies were conducted in accordance with the local legislation and institutional requirements. The participants provided their written informed consent to participate in this study.

## Author contributions

AM: Conceptualization, Supervision, Writing – original draft, Writing – review & editing. TA: Formal analysis, Investigation, Supervision, Writing – original draft, Writing – review & editing. AB: Investigation, Software, Writing – original draft. AD: Supervision, Validation, Writing – review & editing. CL: Validation, Writing – review & editing. FB: Data curation, Methodology, Supervision, Writing – review & editing. PS: Conceptualization, Supervision, Writing – review & editing. MP: Conceptualization, Supervision, Validation, Writing – review & editing.
